# Haemoadsorption Combined with Continuous Renal Replacement Therapy in Abdominal Sepsis: Case Report Series

**DOI:** 10.3390/jpm13071113

**Published:** 2023-07-10

**Authors:** Fernando Sánchez-Morán, María Lidón Mateu-Campos, Francisco Bernal-Julián, Ali Gil-Santana, Ángeles Sánchez-Herrero, Teresa Martínez-Gaspar

**Affiliations:** 1 Intensive Care Unit, General University Hospital of Castellon, Avenida de Benicasim 128, 12004 Castellón de la Plana, Spain; 2 Clinical Analysis Service, General University Hospital of Castellon, Avenida de Benicasim 128, 12004 Castellón de la Plana, Spain

**Keywords:** abdominal sepsis, septic shock, haemoadsorption, cytokines

## Abstract

In recent decades, multiple efforts have been made to identify targets and therapeutic measures in the host response to infection. Haemoadsorption, under the attractive theoretical premise of inflammatory response modulation through the adsorption of soluble inflammatory mediators, could have a place as an adjuvant therapy in septic patients. The development of new devices and the recent COVID-19 pandemic has renewed interest in this therapy. The aim of this report is to describe our experience in patients with abdominal sepsis for whom haemoadsorption with a neutral microporous resin column was added to conventional treatment and to describe its performance through patient cases in the absence of large randomised trials with this device. We present five patients with abdominal sepsis admitted to a Spanish intensive care unit in which haemoadsorption was used as adjuvant treatment. The key practical aspects of the treatment protocol have been used as a guide for conducting a multicentric study. Based on the experience gathered in these five cases, the potential benefit of haemoadsorption as adjuvant therapy in patients with abdominal sepsis with multiple organ failure after control of the source of infection and adequate treatment should be investigated. Likewise, it must be defined which patients can benefit from the therapy, the most appropriate biomarkers to guide the therapy, the ideal time of initiation and discontinuation, its potential side effects, and the interaction with other therapies, especially how such treatment affects the antibiotics levels.

## 1. Introduction

Haemoadsorption is an extracorporeal blood purification (EBP) technique that allows the removal of unwanted plasma solutes by direct adsorption. This technique is based on mass separation by a solid agent (sorbent) contained in a cartridge. Its potential role in sepsis has been largely investigated under the attractive approach of direct elimination of soluble mediators produced during the immune response to the pathogen, especially the potential benefits of lowering cytokine levels [1]. Conversely, most of the studies carried out in sepsis were performed without the measurement of cytokines in very heterogeneous populations.

HA cartridges (Jafron Medical, Zhuhai, China) are classified as nonselective adsorption devices. The cartridges contain neutro-macroporous resin adsorbing beads made of styrene-divinylbenzene copolymer. The Jafron HA380 cartridge has been designed for use in clinical conditions characterised by elevated cytokine levels, such as sepsis and other cytokine release syndromes [2]. The resin pore size distribution ranges from 500 Da to 60 kDa and allows the removal of molecules from 10 kDa to 60 kDa [3].

HA cartridges have been studied in randomised clinical trials combined with conventional treatment in septic shock [4,5,6]. These trials have reported different benefits on haemodynamics, markers of lung injury, duration of mechanical ventilation and continuous renal replacement therapy (CRRT), lower intensive care unit (ICU) length of stay, and an effective reduction in cytokine levels; one of the trials revealed a significant effect on mortality [6].

This report provides the protocol description and the outcomes of five cases of abdominal septic shock and multiple organ failure consecutively treated with HA380 as adjuvant therapy, where a combination of biomarkers, including interleukin 6 (IL-6), and clinical parameters were used as criteria for selection, initiation, or cessation of the therapy.

## 2. Materials and Methods

Five patients with abdominal septic shock developed multiorgan dysfunction syndrome (MODS) despite being treated following the Surviving Sepsis Guidelines [7], where haemoadsorption was added as adjuvant treatment. All patients met the following conditions: (1) controlled abdominal source of infection, (2) norepinephrine > 0.5 μg/kg/min to maintain adequate organ perfusion after optimisation of fluid therapy, (3) dysfunction of two or more organs with a Sepsis-related Organ Failure Assessment (SOFA) score ≥ 9, (4) blood lactate ≥ 2 mmol/L, (5) procalcitonin (PCT) > 10 ng/mL, (6) C-reactive protein (CRP) > 100 mg/L, and (7) interleukin 6 (IL-6) > 2000 pg/mL. Since haemoadsorption cannot replace or delay any intervention in the management of bacterial-induced sepsis, if all seven of the aforementioned criteria were met, haemoadsorption was added to treatment within 12–24 h after adequate control of the source of infection. Patient demographic and clinical data are described in Table 1.

Haemoadsorption was performed with CRRT in continuous venovenous haemodialysis mode (CVVHD) with citrate anticoagulation.

An immunochemiluminescence assay (Cobas^®^ Pro, Roche Diagnostics International Ltd., Rotkreuz, Switzerland) was used to measure IL-6 levels. If the patient met the first six aforementioned requirements, blood samples were taken for the assessment of this mediator for follow-up and therapy guidance after each haemoadsorption session and before the following session. When haemoadsorption was stopped, another sample was taken 24 h later for follow-up to ensure that there was no rebound effect [8].

The HA380 cartridge was placed post-filter. The cartridge was primed with heparin and saline solution in accordance with the manufacturer’s instructions prior to being connected to the circuit. Four patients had CRRT with haemoadsorption added to it, and in one patient, the connection to the CRRT circuit was made exclusively for haemoadsorption. The first patient underwent four consecutive haemoadsorption sessions in three days (two sessions lasting 12 h and two lasting 24 h). For the remaining patients, the frequency and number of sessions depended on whether the clinical and analytical goals (reduction in vasopressors by at least 50% and decrease in IL-6 levels below 1000 pg/mL) were met. None of the following four patients needed more than two sessions. The scheduled time for each session was met as no coagulation of the haemoadsorption cartridge was recorded.

## 3. Results

Clinical and laboratory parameters before and after the treatment are described in Table 2. Before and after the first and second haemoadsorption sessions, IL-6 levels were 4896.80 (±517.47) pg/mL, 1485.00 (±714.36) pg/mL, and 407.60 (IQR 320.75–1125.05) pg/mL, respectively. The mean decrease in IL-6 levels with the first session was 69.72% (±13.90), and after the second session, the median decrease was 91.88% (IQR 77.50–92.94). Along with these findings, there was a reduction in vasopressors from 0.68 (±0.13) μg/kg/min of norepinephrine before treatment to 0.50 (±0.12) and 0.28 (±0.19) after the first and second cartridge, respectively. This means a reduction of 23.91% (±22.90) and 87.23% (±19.20) after the first and second cartridge, respectively. There was also a progressive correction of blood pH with these improvements in haemodynamics. Clinical and laboratory parameters before and after treatment are described in Table 2. All five patients were discharged alive from the ICU and from the hospital.

## 4. Discussion

**Figure 1:**
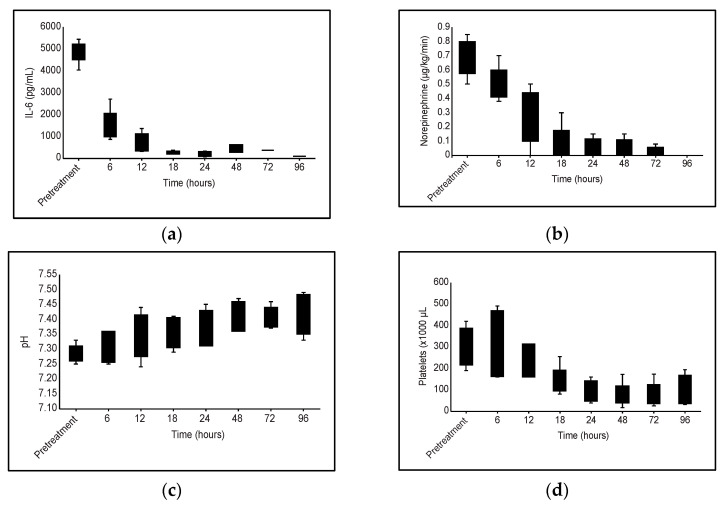
Evolution of different clinical and analytical parameters during haemoadsorption treatment with HA380 cartridge: (**a**) IL-6 levels; (**b**) Vasopressor requirement; (**c**) pH; (**d**) Platelet variation.

The use of haemoadsorption with HA380 added to conventional therapy might have improved haemodynamic stability and actual control of inflammation, as we observed a significant reduction in vasopressor doses and an improvement in organ dysfunction, along with a reduction in IL-6 serum levels. The transitory reduction in platelets is a widely known side effect of haemoadsorption. We cannot rule out other circumstances that could also favour this thrombocytopenia (Figure 1), such as the septic process itself or the possible effect of CRRT programming, but the relationship with haemoadsorption is clear in our patients.

Haemoadsorption could have an impact on antibiotic levels, and it is essential, as in any septic patient, to have a protocol for monitoring the levels of certain antibiotics to facilitate their proper dosing. In one patient in this series, the linezolid dosage regimen was modified, increasing the dose from 600 mg every 12 h to 600 mg every 8 h. In the rest of the patients in the series, no modification was necessary. Despite this finding, we recommend close monitoring of those drugs, especially antibiotics, that could be affected by any blood purification technique or that must be adjusted according to renal function.

EBP therapies have been proposed for many years as a potential treatment for sepsis and septic shock in critically ill patients under the hypothesis that cytokine level stabilisation would improve patient outcomes [9]. There is still controversy over the efficacy of these devices, as many studies have been negative, and the evidence supporting their usage is relatively uncertain or ambiguous [10,11].

The significant heterogeneity of the patient populations included in the clinical trials conducted in sepsis is largely responsible for the unfavourable outcomes [12]. Sepsis has a wide range of clinical characteristics, and part of the heterogeneity can be explained by deregulation of host responses depending on the source of infection. There are stronger abnormalities of the host response in different pathophysiological domains, such as the inflammatory response, activation of endothelial cells, or coagulation, depending on the source of infection [13]. To identify the clinical subphenotypes that might benefit from haemoadsorption, future research should concentrate on selected homogenous populations using biomarkers and probably phenotype profiles [14].

Clinical studies’ primary outcomes should focus on clinical and analytical parameters to evaluate the effects of haemoadsorption on surrogate endpoints other than mortality, such as haemodynamic stabilisation, reduction in serum inflammatory mediators, improvement in organ failure or the reduction in organ support requirements. However, for a trustworthy evaluation of the clinical impact of haemoadsorption, survival should be included as an outcome in large and rigorous randomised controlled trials [10,12].

The ideal biomarker to guide these therapies is still elusive. Although knowledge about the pathophysiology and inflammation in sepsis has evolved in recent decades, we still far from completely understand the connections of the different pathways involved. Some authors suggest that damage-associated molecular patterns (DAMPs), such as high mobility group Box 1 protein (HMGB1) or histones and glycans, could be interesting targets for EBP given the significant mediation of these molecules in systemic inflammation and other pathophysiological domains [15]. Cytokine physiology and their interactions are poorly understood, and little is known about the impact of certain drugs routinely used in sepsis treatment on cytokines [16,17]. It would definitely make sense to distinguish between inflammation and hyperinflammation, as well as between these and the cytokine storm [16,18]. One key point is to determine the threshold of inflammation at which action should be taken if necessary [19].

In our series, we set a level of IL-6 in combination with other biomarkers and clinical conditions to start, guide, and stop the therapy. This approach allowed us to conduct a secure and targeted treatment. The key practical aspects of the treatment protocol have been used as a guide for a multicentric study approved by the Drug Research Ethics Committee of the General University Hospital of Castellon (dREC acta 5/2022) that is being conducted in 6 ICUs in Spain (ClinicalTrials.gov Identifier: NCT05044403).

The aim of this pilot study is to clarify whether the application of haemoadsorption in addition to the current clinical practice can improve organ dysfunction and the requirements of organ support in patients with abdominal septic shock.

Obviously, our report has many limitations. First, it is a small case series. Second, the use of IL-6, may be an oversimplification of the inflammatory process, since it is probably not the most indicated biomarker. Given the kinetics of IL-6 and the timing of initiation of haemoadsorption, we cannot be sure that this cytokine was not in a declining phase. Third, as there is no control group, we cannot rule out the fact that the patients could have improved anyway regardless of the initiation or not of the blood purification therapy in case we were in that declining phase mentioned above. Finally, some data were recorded retrospectively. And although it is not possible to extrapolate valid conclusions from the data obtained given the limitations of our study, we believe our approach might be attractive for the reader interested in the subject, as it might help to generate new questions and hypotheses about the potential role of haemoadsorption in septic shock.

In our opinion, haemoadsorption could be helpful in well-selected populations of patients suffering of demonstrated acute inflammatory disorders such as sepsis, pancreatitis, acute respiratory distress syndrome (ARDS), burns, or pancreatitis; however there still remain a lot of unanswered questions. In order to determine the goals for the therapy, we need to identify the patient population—or subpopulation of patients—who may benefit from extracorporeal blood purification approaches based on adsorption. The right biomarker, or group of biomarkers, must be found in order to establish a threshold that will trigger and direct the therapy. The proper time, dosage, and number of cycles of hemoperfusion have not been studied, nor have the potential negative effects on solutes we do not want to be removed, such as antibiotics or non-antimicrobial drugs.

## 5. Conclusions

Haemoadsorption with HA380 was associated with an effective reduction in IL-6 and with clinical improvement, as shown by the reduction in vasoactive support and haemodynamic stabilisation. The potential benefit of haemoadsorption should be investigated in carefully designed randomised controlled studies in targeted populations to warrant the optimal timing of initiation, duration of therapy and defined endpoints.

## Figures and Tables

**Table 1:** Patient demographic and clinical data.

	Case 1	Case 2	Case 3	Case 4	Case 5
Gender	Male	Female	Male	Female	Female
Age (years)	79	54	29	78	75
Source of infection	Ileal dehiscence	Rectal perforation	Sigmoid perforation	Colonic perforation	Caecum perforation
Cultures					
Blood	-	-	-	*Eggerthella lenta*	-
Peritoneal	*Escherichia coli*	-	*Escherichia coli*	-	*Escherichia coli*
	*Enterococcus faecalis*		*Proteus mirabilis*		*Bacteroides distasonis*
			*Bacteroides fragilis*		*Klebsiella pneumoniae*
					*Pseudomonas aeruginosa*
Antibiotics	Meropenem Linezolid	Meropenem Linezolid	Meropenem Linezolid	Meropenem Linezolid	Meropenem Linezolid
SAPS 3	88	64	50	64	75
SOFA	11	9	10	9	9
Mechanical ventilation	Yes	Yes	Yes	Yes	Yes
AKI	Yes	Yes	Yes	Yes	Yes
KDIGO class	3	3	3	2	3
RRT	Yes	Yes	Yes	No	Yes

AKI: Acute kidney injury; KDIGO: Kidney Disease: Improving Global Outcomes); RRT: Renal replacement therapy; SAPS: Simplified Acute Physiology Score; SOFA: Sepsis-related Organ Failure Assessment.

**Table 2:** Clinical and laboratory parameters before and after treatment with the HA380 cartridge.

Case	Case 1		Case 2		Case 3		Case 4		Case 5	
Initial CRRT dose (mL/kg/h)	29		16		23		22		14	
Cartridges (n)	4		2		2		2		2	
Treatment schedule (hours)	12-12-24-24		6-6		6-6		6-6		6-6	
	Pre	Post	Pre	Post	Pre	Post	Pre	Post	Pre	Post
Urea (mg/dL)	89	54	76	92	129	85	60	53	86	81
Creatinine (mg/dL)	1.51	0.54	2.39	2.26	2.93	0.81	1.21	1.19	2.47	1.46
Potassium (mmol/L)	4.37	3.06	4.1	4.15	4.63	3.38	4.1	2.81	5.65	3.73
Sodium (mmol/L)	140	144	142	135	143	143	143	138	132	127
Bicarbonate (mmol/L)	17.5	28.4	22.1	22.4	27.9	27.9	20.2	27.8	18.4	19.6
Urine volume (mL/24 h)	0	2860	1395	5050	1220	2435	20	4240	333	1440
Hb (g/dL)	10.3	8.9	10.2	8.4	8.4	8.5	14.6	9.7	10.7	7.2
Total white cells (×10^3^/L)	13.42	28	47.55	22.38	16	14.2	5.6	9.2	4	33.8
Platelets (×10^3^/L)	192	64	160	56	491	172	164	60	446	18
PT (s)	16.4	13.7	14	13.8	12	10.8	12.6	11.7	11.1	9.7
aPTT (s)	56.4	34.5	26.6	32.4	24.2	22.8	32.1	39.4	28.6	32.7
Heart rate (beat/mi)	110	68	120	101	110	84	99	84	93	82
MAP (mmHg)	69	93	69	96	76	81	79	81	90	83
Lactate (mmol/L)	5.1	1.7	2.1	0.7	2.0	1.1	6.8	2.0	4.4	1.4
Noradrenaline (μg/kg/min)	0.85	0	0.75	0	0.5	0	0.65	0.07	0.65	0.15
PaO_2_/FiO_2_	158	187	308	520	220	351	206	255	326	420
PCT (ng/mL)	11.49	0.93	36.12	41.86	19.4	3.59	39.91	11.5	35.1	7.74
CRP (mg/L)	487.63	173.2	163	342	411	230	175	281	412.81	205
IL-6 (ng/L)	5000	193	4038	112	5446	216	5000	372	5000	187

CRRT: Continuous renal replacement therapy; Pre: Pretreatment; Post: Posttreatment; Hb: Haemoglobin; PT: Prothrombin time; aPTT: Activated partial thromboplastin time; MAP: Mean arterial pressure; PaO_2_/FiO_2_: Ratio partial pressure of oxygen in alveoli to fraction of inspired oxygen; PCT: Procalcitonin; CRP: C-Reactive protein; IL-6: Interleukin-6.

## Data Availability

Data supporting the results reported in the article can be found by academic researchers, under reasonable request, by sending an email to the corresponding author at sanchez_fermor@gva.es.
